# Supplementary material for: Development and Validation of a Non-Targeted Screening Method for Most Psychoactive, Analgesic, Anaesthetic, Anti-Diabetic, Anti-Coagulant and Anti-Hypertensive Drugs in Human Whole Blood and Plasma Using High-Resolution Mass Spectrometry
Source: Pharmaceuticals (Basel). 2023 Jan 4;16(1):76. doi: 10.3390/ph16010076 (PMC9865759; doi:10.3390/ph16010076)
Supplement: Supplementary file 1 [file pharmaceuticals-16-00076-s001.zip › pharmaceuticals-2079607-supplementary.pdf]

Supplementary file table S1: Validation parameters for the 53 selected compounds: mean and relative standard deviation (RSD, %) for matrix effect (ME), recovery (RE) and process efficiency (PE) (n=6), in both plasma (A) and whole blood (B).

A. Plasma

Compounds	Matrix effect (ME)		Recovery (RE)		Process efficiency (PE)	
	mean	RSD (%)	mean	RSD (%)	mean	RSD (%)
Acetazolamide	0.96	8.23	1.11	3.63	0.86	6.67
Alprazolam	1.25	12.07	0.91	10.78	1.40	18.42
Amiodarone	1.21	7.49	1.05	5.75	1.15	9.99
Amitriptyline	1.06	12.19	1.08	4.00	0.98	12.94
Amlodipine	1.21	17.07	0.97	6.62	1.26	16.44
Aripiprazol	0.95	7.96	1.06	4.66	0.89	8.53
Atenolol	1.28	17.13	1.08	4.33	1.19	18.58
Atropine	0.89	8.80	1.06	4.36	0.85	8.20
Baclofen	1.74	6.80	1.22	6.07	1.43	9.45
Bisoprolol	0.95	6.25	1.08	4.15	0.89	5.60
Bromazepam	1.41	9.49	0.99	9.27	1.45	17.47
Bupivacaine	0.99	5.18	1.08	3.62	0.92	2.85
Buprenorphine	1.04	10.29	1.06	6.62	0.98	11.96
Citalopram	0.98	8.82	1.08	4.78	0.90	8.29
Clomipramine	0.95	11.67	1.10	5.28	0.86	10.54
Clonazepam	1.23	19.94	1.10	7.95	1.13	19.23
Clozapine	1.34	14.91	1.11	5.53	1.20	13.52
Cocaine	1.04	8.01	1.05	6.01	0.99	9.01
Cyamemazine	0.96	8.45	1.06	8.01	0.90	10.38
Diazepam	0.97	18.81	1.13	5.67	0.86	15.12
Fentanyl	1.06	8.14	1.06	4.77	1.00	10.14
Flupentixol	1.07	9.42	1.05	7.10	1.02	6.72
Furosemide	1.15	18.89	1.19	10.30	0.96	11.17
Glibenclamide	1.36	19.65	1.11	6.83	1.24	13.59
Haloperidol	1.05	10.77	1.09	7.78	0.97	14.06
Hydroxyzine	1.04	13.19	1.08	4.90	0.96	11.64
Ketamine	1.21	13.05	1.05	4.51	1.15	13.03
Lamotrigine	0.98	11.83	1.13	7.42	0.87	11.10
Levetiracetam	1.02	6.74	1.06	5.65	0.96	8.10
Lidocaine	1.15	11.04	1.06	3.75	1.08	9.77
Loxapine	1.25	13.58	1.11	5.55	1.14	16.00
MDA	1.54	9.86	1.10	3.91	1.40	12.36
Metformin	0.15	18.06	1.16	4.94	0.12	16.34
Methadone	1.14	9.79	1.05	6.26	1.08	10.54
Mianserin	1.05	12.28	1.09	3.66	0.96	12.07
Midazolam	0.94	5.29	1.16	13.26	0.65	10.86
Mirtazapine	1.26	14.75	1.10	9.33	1.16	18.93
Morphine	2.28	13.41	1.13	3.83	2.01	13.49
Nefopam	1.10	10.30	1.09	5.12	1.00	11.28
Nordiazepam	1.10	10.30	1.09	5.12	1.00	11.28
O-demethylvenlafaxine	1.01	8.64	1.07	3.10	0.94	8.29
Oxazepam	1.58	16.00	1.09	9.25	1.47	18.59
Paroxetine	0.95	10.22	1.09	7.84	0.88	8.87

Prazepam	0.91	19.01	1.05	4.70	0.86	15.05
Propranolol	1.01	6.76	1.08	3.97	0.94	5.38
Risperidone	2.01	26.19	1.11	6.72	1.81	14.51
Sertraline	0.86	16.08	1.11	5.19	0.78	13.54
Tiapride	0.99	9.40	1.06	4.28	0.94	9.00
Tramadol	1.10	8.88	1.06	5.94	1.04	8.63
Venlafaxine	1.07	10.54	1.09	5.44	0.98	11.51
Verapamil	0.99	7.68	1.04	4.77	0.95	7.47
Warfarin	1.22	9.98	1.11	9.20	1.10	14.80
Zolpidem	1.06	10.49	1.13	9.67	0.94	13.61

B. Whole Blood

Compounds	Matrix effect (ME)		Recovery (RE)		Process efficiency (PE)	
	mean	RSD ()	mean	RSD ()	mean	RSD ()
Acetazolamide	0.92	8.65	1.15	12.90	0.80	14.77
Alprazolam	0.88	5.62	0.70	19.85	1.25	19.96
Amiodarone	0.96	2.28	1.15	15.39	0.83	15.34
Amitriptyline	1.04	16.60	1.19	17.19	0.87	17.79
Amlodipine	1.12	10.64	1.14	14.69	0.99	11.86
Aripiprazol	0.94	10.20	1.09	16.77	0.87	19.92
Atenolol	1.93	14.66	1.15	5.12	1.68	17.08
Atropine	0.92	15.21	1.17	15.64	0.79	18.31
Baclofen	1.29	18.86	1.15	7.90	1.12	18.60
Bisoprolol	1.10	11.70	1.19	7.04	0.93	13.94
Bromazepam	1.08	16.47	1.09	19.83	1.00	16.85
Bupivacaine	0.93	14.54	1.19	17.30	0.78	18.00
Buprenorphine	0.99	19.50	1.11	13.70	0.89	19.17
Citalopram	0.99	9.70	1.15	13.99	0.86	18.42
Clomipramine	0.95	13.12	1.14	16.18	0.83	17.27
Clonazepam	1.05	17.09	1.13	16.65	0.93	16.76
Clozapine	1.15	10.61	1.15	9.46	1.00	17.28
Cocaine	1.02	16.86	1.17	13.72	0.87	17.73
Cyamemazine	0.93	11.55	1.02	9.78	0.92	14.35
Diazepam	1.02	18.39	1.10	11.31	0.93	18.34
Fentanyl	1.02	16.47	1.20	18.17	0.85	18.64
Flupentixol	1.01	13.75	0.93	17.88	1.09	19.61
Furosemide	1.06	5.83	1.11	18.01	0.96	15.99
Glibenclamide	0.83	10.47	1.05	19.25	0.79	16.34
Haloperidol	0.98	16.25	1.08	10.96	0.91	17.67
Hydroxyzine	1.04	13.20	1.17	19.37	0.89	18.01
Ketamine	1.20	17.90	1.16	12.52	1.03	16.33
Lamotrigine	1.00	15.41	1.15	11.02	0.87	15.40
Levetiracetam	1.01	9.21	1.17	11.61	0.87	11.81
Lidocaine	1.15	16.28	1.18	10.99	0.98	16.90
Loxapine	1.20	19.69	1.22	19.52	0.98	19.07
MDA	1.68	14.42	1.23	5.28	1.37	17.42
Metformin	0.15	12.96	1.30	13.15	0.11	24.98
Methadone	1.10	19.14	1.17	16.94	0.94	17.68
Mianserin	1.06	14.47	1.21	19.53	0.84	17.22
Midazolam	0.92	19.24	1.11	12.60	0.83	14.05

Mirtazapine	1.24	19.45	1.12	6.95	1.11	13.83
Morphine	2.17	18.33	1.18	19.64	1.84	14.51
Nefopam	1.06	17.93	1.15	11.68	0.92	15.40
Nordiazepam	1.24	17.58	1.00	17.77	1.24	19.70
O-demethylvenlafaxine	1.04	11.28	1.10	5.79	0.95	14.53
Oxazepam	1.19	11.87	1.08	19.41	1.10	16.03
Paroxetine	0.98	17.75	1.16	11.75	0.85	16.59
Prazepam	0.88	11.64	1.08	10.17	0.82	16.23
Propranolol	0.99	17.45	1.10	10.94	0.90	16.51
Risperidone	1.52	15.58	1.18	18.32	1.29	16.99
Sertraline	0.75	16.13	1.05	15.51	0.71	18.26
Tiapride	0.75	16.13	1.13	5.98	0.66	19.84
Tramadol	1.18	14.32	1.13	6.43	1.04	12.19
Venlafaxine	1.02	18.91	1.14	12.75	0.90	14.91
Verapamil	0.93	19.90	1.14	19.68	0.82	17.78
Warfarin	1.04	11.93	1.20	19.47	0.87	19.80
Zolpidem	1.02	18.11	1.15	6.04	0.88	13.91

Compounds	Helfer *et al*	Roche *et al*	Joye *et al*	The present assay
	LOI (µg/L) in plasma (precipitation/turboflo w)	LOI (µg/L) in plasma/whole blood	LOI (µg/L) in dried blood spot	LOI (µg/L) in plasma/whole blood
6-monoacetylmorphine	NA	NA	5	0.98/0.98
7 aminoclonazepam	NA	NA	10	0.78/0.78
9-hydroxyrisperidone	NA	NA	NA	0.5/1
acebutolol	NA	NA	NA	1/1
N-acetyl-acebutolol	NA	NA	NA	1/1
acenocoumarol	NA	NA	NA	5/5
acepromazine	NA	NA	NA	0.5/5
acetaminophen	NA	55.6/60.1	NA	NA
acetazolamide	NA	NA	NA	100/100
alimemazine	NA	NA	NA	0.5/1
aliskiren	10/10	NA	NA	NA
alprazolam	NA	NA	NA	0.78/1.56
altizide	NA	NA	NA	10/10
amiloride	NA	NA	NA	10/10
amiodarone	1000/100	NA	NA	10/10
amisulpride	NA	NA	NA	0.5/0.5
amitriptyline	1/1	NA	1	0.5/0.5
amlodipine	10/1	NA	NA	5/5
amphetamine	100/10	NA	10	1.95/1.95
anhydromethylecgonine	NA	NA	NA	7.81/7.81
aripiprazole	NA	NA	NA	1/1
atenolol	NA	2.3/0.9	NA	10/1
atropine	NA	NA	NA	1/10
baclofene	NA	NA	NA	40/40
bendroflumethiazide	NA	NA	NA	10/10
benzoylecgonine	NA	NA	1	0.98/0.98
benzylpiperazine	NA	NA	1	NA
bezafibrate	1000/10	NA	NA	NA
bisoprolol	10/0.1	NA	NA	1/1
bromazepam	NA	NA	NA	1.98/7.81
bumetanide	NA	NA	NA	10/10
bupivacaine	NA	NA	NA	1/1
buprenorphine	NA	NA	NA	1/1
bupropion	NA	NA	NA	0.5/0.5
butylone	NA	NA	1	NA
canrenone	NA	NA	NA	10/10
carbamazepine	NA	NA	1	NA
cefazoline	NA	NA	NA	2000/2000
cefoxitine	NA	NA	NA	400/400
ceftazidime	NA	NA	NA	400/400

celiprolol	NA	NA	NA	1/1
chlordiazepoxide	NA	NA	NA	0.99/0.99
chlorophacinone	NA	NA	NA	50/50
chloroquine	NA	NA	NA	20/20
chlorpromazine	NA	NA	NA	2/1
ciprofloxacine	NA	NA	NA	20/20
citalopram	NA	NA	1	0.5/0.5
clindamycine	NA	NA	NA	20/20
clobazam	NA	NA	NA	0.99/1.98
clomipramine	NA	NA	NA	1/2
clonazepam	NA	NA	NA	25/25
clotiazepam	NA	NA	NA	0.99/1.98
clozapine	NA	1.6/0.6	NA	0.5/0.5
cocaethylene	NA	NA	NA	0.98/0.98
cocaine	NA	NA	1	0.98/0.98
codeine	10/10	NA	5	3.91/3.91
coumatetralyl	NA	NA	NA	50/50
cyamemazine	NA	NA	NA	1/0.5
declomipramine	NA	NA	NA	0.5/0.5
demethylcitalopram	NA	NA	NA	0.5/0.5
desipramine	NA	NA	NA	1/2
desmethylnefopam	NA	NA	NA	0.5/0.5
desmethylzopiclone	NA	NA	NA	1.56/1.56
dextrometorphane	NA	NA	NA	0.98/0.98
diazepam	1/0.1	1.7/1	1	0.99/0.99
diclofenac	1000/100	NA	NA	1/1
didemethylcitalopram	NA	NA	NA	50/50
difenacoum	NA	NA	NA	50/50
dihydrocodeine	NA	NA	NA	0.98/0.98
diltiazem	NA	NA	NA	0.5/0.5
diphenandione	NA	NA	NA	50/50
diphenhydramine	1/0.1	NA	NA	NA
dosulepine	NA	NA	NA	0.5/0.5
doxazosin	1/1	NA	NA	NA
doxepine	NA	NA	NA	0.5/0.5
estazolam	NA	NA	NA	0.99/0.99
ethylmorphine	NA	NA	NA	1.95/1.95
fentanyl	NA	0.4/0.3	NA	1/1
flecaine	NA	NA	NA	1/1
fluconazole	NA	NA	NA	500/500
flufenamic acid	1000/1000	NA	NA	NA
fluindione	NA	NA	NA	500/500
fluoxetine	NA	NA	1	NA
flupentixol	NA	NA	NA	0.5/0.5

fluphenazine	NA	NA	NA	0.5/0.5
fluvoxamine	NA	NA	NA	1/2
furosemide	NA	NA	NA	10/10
gabapentine	NA	NA	20	NA
glibenclamide	NA	NA	NA	2.5/5
gliclazide	NA	NA	NA	5/5
glimepiride	NA	NA	NA	10/10
glipizide	NA	NA	NA	5/5
haloperidol	NA	1.6/0.6	1	0.5/0.5
heroine	NA	NA	NA	0.98/0.98
hydrochlorothiazide	100/10	NA	NA	NA
hydroxychloroquine	NA	NA	NA	20/20
hydroxy-Itraconazole	NA	NA	NA	200/200
hydroxymidazolam	NA	NA	1	NA
hydroxyzine	NA	NA	NA	0.99/0.99
imipramine	NA	1.7/0.7	NA	0.5/0.5
indapamide	NA	NA	NA	10/10
itraconazole	NA	NA	NA	200/200
ivabradine	1/0.1	NA	NA	NA
ketamin	NA	NA	5	10/10
labetalol	NA	NA	NA	1/1
lamotrigine	NA	NA	NA	10/10
levetiracetam	10/10	NA	NA	100/100
levomepromazine	NA	NA	NA	0.5/1
lidocaine	NA	NA	NA	1/1
linezolide	NA	NA	NA	100/100
loprazolam	NA	NA	NA	3.13/3.13
lorazepam	NA	NA	NA	3.13/12.5
lormetazepam	NA	NA	NA	3.13/12.5
Losartan	10/1	NA	NA	NA
loxapine	NA	NA	NA	0.5/0.5
LSD	NA	NA	NA	0.39/0.39
maprotiline	NA	NA	NA	0.5/0.5
MBDB	NA	NA	NA	0.98/0.98
MDA	NA	NA	NA	6.25/6.25
MDEA	NA	NA	NA	0.98/0.98
MDMA	NA	NA	1	1.95/1.95
mephedrone	NA	NA	NA	1.95/1.95
mepivacaine	NA	NA	NA	1/1
mequitazine	NA	NA	NA	0.5/0.5
meropenem	NA	NA	NA	500/500
metamphetamine	NA	1.8/1	1	0.98/0.8
metformine	NA	NA	NA	100/100
methadone	NA	NA	1	0.98/0.98

methedrone	NA	NA	1	NA
methylclothiazide	NA	NA	NA	10/10
methylecgonine	NA	NA	NA	62.5/62.5
methylone	NA	NA	1	0.98/0.98
methylprednisolone	NA	NA	NA	10/20
metoclopramide	NA	NA	NA	0.5/1
metoprolol	NA	NA	NA	10/10
mianserine	NA	NA	1	0.5/0.5
midazolam	NA	1.1/2.6	1	0.99/0.99
milnacipran	NA	NA	NA	0.5/0.5
minoxidil	1/1	NA	NA	NA
mirtazapine	NA	NA	NA	0.5/0.5
moclobemide	NA	NA	NA	0.5/0.5
molsidomine	1/1	NA	NA	NA
morphine	100/10	0.9/1.3	10	10/10
moxonidine	1/1	NA	NA	NA
nadolol	NA	NA	NA	1/1
nalbuphine	NA	NA	NA	0.98/0.98
naloxone	NA	NA	NA	0.98/0.98
nefopam	NA	NA	NA	0.5/0.5
nicardipine	NA	NA	NA	0.5/0.5
nifedipine	NA	NA	NA	2/2
nitrazepam	NA	NA	NA	0.78/6.25
norbuprenorphine	NA	NA	NA	3.13/3.13
norclobazam	NA	NA	NA	4.9/9.8
norclozapine	NA	NA	NA	0.5/0.5
norcodeine	NA	NA	NA	1.95/1.95
norcyamemazine	NA	NA	NA	0.5/1
nordiazepam	NA	NA	NA	0.99/1.95
nordoxepine	NA	NA	NA	0.5/0.5
norLSD	NA	NA	NA	0.39/0.39
norpropoxyphene	NA	NA	NA	1.95/1.95
nortriptyline	NA	NA	NA	1/0.5
N-demethylvenlafaxine	NA	NA	NA	0.5/0.5
O-demethylvenlafaxine	NA	NA	NA	1/1
ofloxacine	NA	NA	NA	20/20
olanzapine	NA	NA	NA	0.5/1
oxazepam	NA	NA	NA	0.99/0.99
oxycodone	NA	NA	NA	0.98/0.98
paroxetine	NA	NA	NA	0.5/0.5
phenobarbital	NA	116.6/88.8	NA	non
phenprocoumon	10/1	NA	NA	0
pholcodine	NA	NA	NA	7.81/7.81
pindolol	NA	NA	NA	1/1

pipamperone	NA	NA	NA	0.5/0.5
pipothiazine	NA	NA	NA	0.5/1
posaconazole	NA	NA	NA	200/200
prazepam	NA	NA	NA	0.99/3.91
prednisolone	NA	NA	NA	10/10
probenicide	NA	NA	NA	10/10
promethazine	1/1	NA	NA	0.5/0.5
propoxyphene	NA	NA	NA	0.98/0.98
propranolol	NA	NA	NA	1/1
quetiapine	1/0.1	NA	1	0.5/1
ramipril	10/10	NA	NA	NA
repaglinide	NA	NA	NA	2.5/2.5
risperidone	1/1	NA	1	0.5/0.5
ropivacaine	NA	NA	NA	1/1
sertraline	1/1	NA	NA	0.5/2
sotalol	NA	NA	NA	1/10
spironolactone	100/10	NA	NA	NA
temazepam	NA	NA	NA	0.99/1.98
THC-COOH	NA	NA	10	NA
thiopental	1000/100	NA	NA	NA
tianeptine	NA	NA	NA	0.5/1
tiapride	NA	NA	NA	1/0.5
timolol	NA	NA	NA	1/1
torasemide	1/1	NA	NA	NA
tramadol	NA	1.7/1.3	NA	1/1
triampterene	0.1/0.1	NA	NA	10/10
trimipramine	NA	NA	5	0.5/1
venlafaxine	NA	1.1/1	NA	0.5/0.5
verapamil	1/0.1	NA	NA	0.5/0.5
voriconazole	NA	NA	NA	200/200
warfarine	NA	NA	NA	10/10
zolpidem	NA	1.3/1.1	1	0.99/0.99
zopiclone	NA	NA	NA	0.78/6.25
zuclopentixol	NA	NA	NA	0.5/1

Supplementary file Table S2: limits of identification (μg/L) for both matrices, with a large panel of 179 compounds and comparison between our assay and previously published studies using UHPLC–HRMS technology

Supplementary file Table S3: Spectra reproducibility for both plasma and whole blood

Compounds	10-fold LOI		3-fold-LOI	
	Mean	RSD (%)	Mean	RSD (%)
Acetazolamide	851.3	2.8	813.3	4.6
Alprazolam	448.5	14.1	417.3	7.3
Amiodarone	903.0	1.3	891.3	1.7
Amitryptyline	750.7	1.1	755.2	0.7
Amlodipine	830.7	1.4	434.5	22.9
Aripiprazol	958.2	1.1	962.0	0.8
Atenolol	778.5	2.9	779.3	2.6
Atropine	719.3	2.1	718.8	4.2
Baclofen	117.3	7.3	127.8	4.8
Bisoprolol	936.3	1.1	938.8	0.6
Bromazepam	732.5	0.8	727.0	2.2
Bupivacaine	380.5	19.5	312.0	14.8
Buprenorphine	525.3	9.6	527.2	4.5
Citalopram	819.6	10.8	799.2	12.3
Clomipramine	316.5	12.7	244.7	8.3
Clonazepam	496.5	14.1	468.2	18.9
Clozapine	699.8	7.1	695.7	3.5
Cocaine	863.0	2.1	864.5	2.4
Cyamemazine	738.3	4.6	757.0	1.3
Diazepam	453.2	3.2	438.5	6.3
Fentanyl	656.7	7.2	636.5	8.5
Flupentixol	920.8	1.7	912.2	1.4
Furosemide	852.2	2.4	822.3	9.5
Glibenclamide	959.3	1.5	943.0	1.2
Haloperidol	619.0	2.4	603.8	6.4
Hydroxyzine	839.2	4.4	829.8	3.6
Ketamine	757.0	2.1	767.5	2.8
Lamotrigine	575.2	6.9	601.3	2.4
Levetiracetam	45.5	1.49	46.7	25.8
Lidocaine	346.5	4.7	323.0	7.2
Loxapine	739.3	6.3	680.7	18.9
MDA	345.3	48.5	403.3	7.1
Metformin	155.7	18.1	142.4	15.2
Methadone	870.8	1.8	869.2	1.3
Mianserine	672.2	1.5	657.3	4.1
Midazolam	710.6	12.5	781.5	2.7
Mirtazapine	347.2	9.5	347.2	5.6
Morphine	338.3	13.4	311.0	6.6
Nefopam	485.3	8.3	428.5	11.7
Nordiazepam	715.2	4.3	707.8	3.9
O-demethylvenlafaxine	561.8	11.1	604.8	7.1
Oxazepam	796.7	1.4	788.8	2.4
Paroxetine	881.2	4.1	853.3	7.0
Prazepam	719.7	16.5	557.0	14.7
Propranolol	821.2	2.6	834.5	4.3
Risperidone	822.3	4.4	791.7	6.9
Sertraline	612.5	15.1	648.7	11.8
Tiapride	776.5	1.7	790.3	1.8
Tramadol	259.3	19.5	246.8	19.8

Trazodone	733.3	1.6	738.5	3.0
Venlafaxine	757.7	3.7	766.5	4.8
Verapamil	960.2	2.2	973.8	0.3
Warfarine	763.7	2.3	782.0	2.5
Zolpidem	541.5	3.7	548.5	2.2

Supplementary table S4: Screening results for internal spiked plasma control (sample 1) and proficiency test (sample 2, 3 and 4).

Sample	Compounds	Spiked concentration (µg.L^{-1})	Therapeutic concentrations (µg.L^{-1}) (45,46)	Identification
1	Amlodipine	10	3-15	Detected
	Atenolol	100	100-2 000	Detected
	Bupivacaine	100	250-200	Detected
	Cocaine	100	50-300	Detected
	Cyamemazine	100	50-400	Detected
	Furosemide	1 000	2 000-5 000	Detected
	Glibenclamide	100	50-200	Detected
	Haloperidol	10	5-17	Detected
	Ketamine	1 000	1 000-6 000	Detected
	Levetiracetam	1 000	3 000-40 000	Detected
	Metformin	500	100-1 300	Detected
	Morphine	100	10-100	Detected
	Prazepam	100	200-700	Detected
	Venlafaxine	100	100-400	Detected
	Warfarine	1 000	1 000-7 000	Detected
2	Baclofen	177	80-400	Detected
	Oxazepam	1 951	200-1 500	Detected
	Citalopram	196	50-110	Detected
	Demethylcitalopram	119	Not available	Detected
	Mephedrone	52	Not available	Detected
3	Amisulpride	Not available	100-320	Detected
	Bisoprolol	450	10-100	Detected
4	Tramadol	2 181	10-1000	Detected
	Rivaroxaban	Not available	Not available	Detected
